# Avian Influenza (H7N9) Viruses Co-circulating among Chickens, Southern China

**DOI:** 10.3201/eid2312.170782

**Published:** 2017-12

**Authors:** Nianchen Wang, Minhua Sun, Wenqing Wang, Guowen Ouyang, Zuxian Chen, You Zhang, Bingbing Zhao, Siyu Wu, Jianni Huang, Hailiang Sun, Ming Liao, Peirong Jiao

**Affiliations:** South China Agricultural University, Guangzhou, China (N. Wang, W. Wang, G. Ouyang, Z. Chen, Y. Zhang, B. Zhao, S. Wu, J. Huang, H. Sun, M. Liao, P. Jiao);; Institute of Animal Health, Guangdong Academy of Agricultural Sciences, Guangdong, China (M. Sun)

**Keywords:** H7N9, avian influenza virus, reassortant, pathogenicity, viruses, China, influenza, respiratory infections

## Abstract

In April 2017, three avian influenza (H7N9) viruses were isolated from chickens in southern China. Each virus had different insertion points in the cleavage site of the hemagglutinin protein compared to the first identified H7N9 virus. We determined that these viruses were double or triple reassortant viruses.

Since its first documentation on March 30, 2013, through March 16, 2017, avian influenza (H7N9) virus has caused 5 epidemic waves of infection among humans in China, resulting in 1,307 laboratory-confirmed clinical cases and 489 deaths (*1*). According to reports of H7N9 virus outbreaks among humans in China, the virus clustered into the Yangtze River Delta lineage and the Pearl River Delta lineage (*2*). As with most low-pathogenicity avian influenza viruses, the early H7N9 avian influenza virus produced mild symptoms in domestic poultry and was therefore generally only detected through active virologic surveillance (*3**,**4*).

In April 2017, H7N9 viruses (isolates A/chicken/Guangdong/Q1/2016, A/chicken/Guangdong/Q26/2017, and A/chicken/Guangdong/Q39/2017, hereafter Q1, Q26, and Q39) were identified from lung samples of chicken, which were collected from Guangdong of China in June 2016 and January 2017. We sequenced all 8 genes of these viruses to trace the origin and clarify the genetic properties. The nucleotide sequences are available from GenBank (accession nos. MF280181–204). 

The H7 hemagglutinin (HA) gene of all 3 viruses belonged to the Yangtze River Delta lineage A (Figure). However, unlike the early H7N9 virus, the HA genes were 1,695 bp and coded 565 aa, and the isolates had 4 inserted amino acids at cleavage sites (KRTAR↓G). In addition, Q26 and Q39 had 4 continuous basic amino acids at cleavage sites (KRKRTAR↓G), which is a characteristic of highly pathogenic avian influenza virus (Technical Appendix Table 1). Q1 had a mutation (Q226L) at the receptor binding site of the HA protein, indicating a higher binding affinity for sialic acid α2,6, a characteristic of human cell-surface receptors (*5*).

**Figure:**
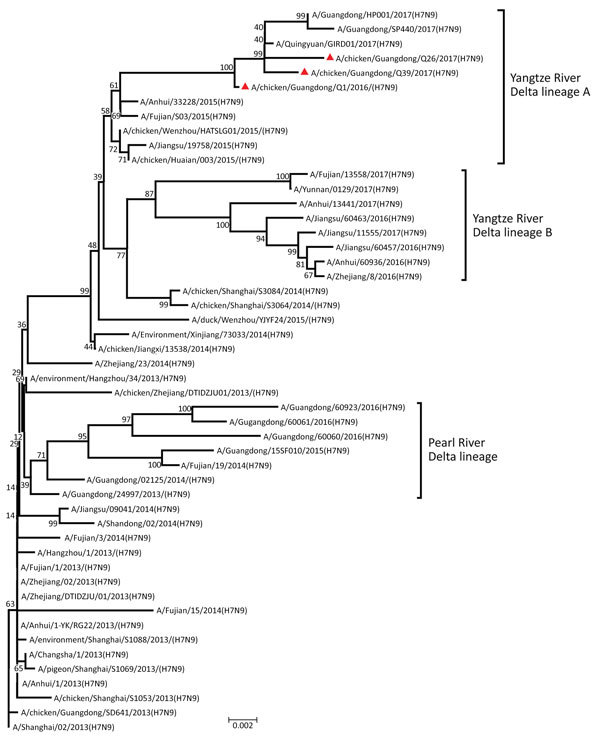
Phylogenetic analysis of the hemagglutinin gene of 3 isolates (triangles) of avian influenza (H7N9) virus obtained from chickens in southern China, 2016–2017, compared with reference isolates. The tree was constructed by using the neighbor-joining method with the maximum composite likelihood model in MEGA version 5.2 (http://www.megasoftware.net) with 1,000 bootstrap replicates, based on the nucleotide sequence 1–1,695. Virus lineages are shown at right. Scale bar indicates nucleotide substitutions per site.

Both Q1 and Q39 had an NA gene of Yangtze River Delta lineage A, whereas the NA gene of Q26 was of Pearl River Delta lineage (Technical Appendix Table 1, Figure). A246T and R292K, which are related to drug resistance, had no substitution in the NA protein of the viruses we analyzed.

The polymerase basic (PB) 1 and 2, polymerase acidic, and nonstructural genes of Q1 and Q39 were all of Yangtze River Delta lineage A, and nucleoprotein genes were of Yangtze River Delta lineage B. The PB2 and nucleoprotein genes of Q26 were of Yangtze River Delta lineage A; PB1, polymerase acidic, and nonstructural genes of Q26 were clustered to the Pearl River Delta lineage (Technical Appendix Table 1, Figure). E627K and D701N had no substitution in the PB2 protein of the viruses, which was thought to contribute to the adaptation, replication, and virulence of influenza viruses in humans and mice (*6*,*7*).

Of particular note, the matrix M gene of Q1 clustered into A/goose/Guangdong/1/96-lineage (H5N1) (GSGD96 lineage) and had a nucleotide of 94.8%. However, the matrix genes of Q26 and Q39 clustered into Yangtze River Delta lineage B of H7N9 virus (Technical Appendix Table 1, Figure).

To clarify the pathogenicity and transmission of the virus, we inoculated 11 chickens with each isolate (10^6^ 50% egg infectious dose [EID_50_] in 0.1 mL of phosphate-buffered saline) and 3 chickens with 0.1 mL phosphate-buffered saline as the control group. We observed all chickens for clinical symptoms for 14 days. The infected chickens exhibited anorexia and signs of depression at 2 days postinoculation (DPI). The Q1 inoculated group died within 4 DPI, Q26 within 3 DPI, and Q39 within 2 DPI; contact groups of Q1, Q26, and Q39 died within 7 DPI, 4 DPI, and 5 DPI, respectively. These viruses replicated systemically in chickens of inoculated groups and were detected in all of the tested organs by 3 DPI. The mean virus titers in the trachea, liver, spleen, kidney, brain, and lung were 4.92–8.42 log_10_ EID_50_/g/0.1 mL (Technical Appendix Table 2). The Q1, Q26, and Q39 virus titers in lung samples for each contact group were 7.5–8.1 log_10_ EID_50_/g/0.1 mL_._ Therefore, the new H7N9 viruses were highly pathogenic to chickens when compared with the early H7N9 virus and could transmit among chickens by contact.

The biological features of H7N9 virus and its pandemic potential have caused global concern (*8*). The early H7N9 viruses lacked the basic HA cleavage site, exhibited low pathogenicity, and caused mild or no disease in poultry (*9*). The cleavage site in HA protein of the isolates we analyzed were KGKRTAR↓G or KRKRTAR↓G. They had high pathogenicity and replication in chickens and could transmit among chickens by contact. Therefore, these new H7N9 viruses could cause a pandemic among poultry and humans in China.

Molecular evolution showed that Q1 was a triple reassortant virus (H5, H7, and H9 subtypes) consisting of Yangtze River Delta A and B lineages of H7N9 and GSGD96 lineage of H5N1. The Q26 and Q39 viruses were both double reassortant avian influenza viruses (H7 and H9 subtype), as was the early H7N9 virus (Figure; Technical Appendix Table 1, Figure). Therefore, the 3 H7N9 viruses we isolated have 2 kinds of insertions in the cleavage sites and were likely derived from different lineages of H7N9 viruses, or even from different subtypes that were co-circulating in southern China during 2016–2017.

Technical Appendix Phylogenetic analysis of avian influenza gene segments, nucleotide identity and phylogenetic lineage of avian influenza virus strains co-circulating among chickens, and characteristics of 3 novel avian influenza H7N9 viruses, China, 2016–2017.
