# Supplementary material for: Avian Influenza (H7N9) Viruses Co-circulating among Chickens, Southern China
Source: Emerg Infect Dis. 2017 Dec;23(12):2100–2. doi: 10.3201/eid2312.170782 (PMC5708235; doi:10.3201/eid2312.170782)
Supplement: Technical Appendix — Phylogenetic analysis of avian influenza gene segments, nucleotide identity and phylogenetic lineage of avian influenza virus strains co-circulating among chickens, and characteristics of 3 novel avian influenza H7N9 viruses, China, 2016–2017. [file 17-0782-Techapp-s1.pdf]

DOI: https://doi.org/10.3201/eid2312.170782

Avian Influenza (H7N9) Viruses Cocirculating Among Chickens, Southern China, 2016–2017

Technical Appendix

Technical Appendix Table 1. Avian influenza viruses cocircluating among chickens in southern China during 2016–2017. The highest nucleotide sequence identity to the new H7N9 AIV was determined by using BLAST search in GenBank*

	Closest viruses in GenBank		
Gene segment	Strain	Nucleotide identity, %	Phylogenetic lineage
HA Q1	A/chicken/Wenzhou/HATSLG01/2015(H7N9)	99.0	YRD A
HA Q26	A/chicken/Wenzhou/HATSLG01/2015(H7N9)	98.5	YRD A
HA Q39	A/chicken/Wenzhou/HATSLG01/2015(H7N9)	98.6	YRD A
NA Q1	A/chicken/Wenzhou/HATSLG01/2015(H7N9)	99.3	YRD A
NA Q26	A/chicken/Shantou/4325/2014(H7N9)	98.9	PRD
NA Q 39	A/chicken/Wenzhou/HATSLG01/2015(H7N9)	98.9	YRD A
PB2 Q1	A/chicken/Zhejiang/925134/2014(H9N2)	99.3	YRD A
PB2 Q26	A/chicken/Zhejiang/SIC40/2015(H9N2)	99.2	YRD A
PB2 Q39	A/chicken/Zhejiang/925134/2014(H9N2)	98.8	YRD A
PB1 Q1	A/chicken/Jiangsu/JS4539/2014(H9N2)	99.2	YRD A
PB1 Q26	A/chicken/Zhejiang/SIC40/2015(H9N2)	98.6	PRD
PB1 Q39	A/chicken/Jiangsu/JS4539/2014(H9N2)	98.9	YRD A
PA Q1	A/chicken/Zhejiang/925134/2014(H9N2)	99.4	YRD A
PA Q26	A/chicken/Shandong/SIC34/2014(H9N2)	98.3	PRD
PA Q39	A/chicken/Zhejiang/925134/2014(H9N2)	99.1	YRD A
NP Q1	A/chicken/Zhejiang/925134/2014(H9N2)	98.3	YRD B
NP Q26	A/chicken/Dongguan/1674/2014(H9N2)	98.8	YRD A
NP Q39	A/chicken/Zhejiang/925134/2014(H9N2)	99.3	YRD B
M Q1	A/chicken/Shenzhen/2396/2013(H5N6)	99.4	GSGD96
M Q26	A/chicken/Zhejiang/SIC40/2015(H9N2)	99.4	YRD B
M Q39	A/chicken/Zhejiang/925134/2014(H9N2)	98.9	YRD B
NS Q1	A/chicken/Zhejiang/925134/2014(H9N2)	99.8	YRD A

	Closest viruses in GenBank		
Gene segment	Strain	Nucleotide identity, %	Phylogenetic lineage
NS Q26	A/chicken/Wenzhou/YHQL04/2014(H9N2)	99.4	PRD
NS Q39	A/chicken/Zhejiang/925134/2014(H9N2)	99.8	YRD A

*http://www.ncbi.nlm.nih.gov/. HA, hemagglutinin; YRD A, Yangtze River Delta A; YRD B, Yangtze River Delta B; NA, neuraminidase; PRD, Pearl River Delta; PB, polymerase basic subunit; PA, polymerase acidic subunit; NP, nucleoprotein; M, matrix; NS, non-structural. GSGD96, A/goose/Guangdong/1/96-lineage (H5N1).

Technical Appendix Table 2. Characteristics of 3 novel avian influenza (H7N9) viruses isolated from chickens in southern China, 2016–2017

		Titer	Virus replication on 3-D post-inoculation (DPI; $log_{10}EID_{50}$/g/0.1ml)* in organ						Contact $log_{10}EID_{50}$/ g/0.1mL
Strain	Characteristic	$Log_{10}EID_{50}$	Liver	Spleen	Kidney	Brain	Trachea	Lung	Lung
Q1	KGKRTAR□G	8.17	5.83 ± 0.58	5.58 ± 0.14	5.67 ± 0.14	4.92 ± 1.23	5.75 ± 0.43	7.42 ± 0.14	7.5
Q26	KRKRTAR□G	7.17	7.67 ± 0.14	7.5 ± 0.75	7.92 ± 0.52	6.75 ± 0.43	7.58 ± 0.14	8.42 ± 0.14	8.1 ± 0.33
Q39	KRKRTAR□G	7.17	7.08 ± 0.52	7.08 ± 0.52	7.75 ± 1.09	6.33 ± 0.14	7.25 ± 0.66	7.83 ± 1.18	8 ± 0.67

*For statistical analysis, a value of 1.5 was assigned if the virus was not detected from the undiluted sample in 3 embryonated eggs. Virus titers were expressed as means standard deviation in log_{10} EID_{50}/g/0.1mL of tissue.

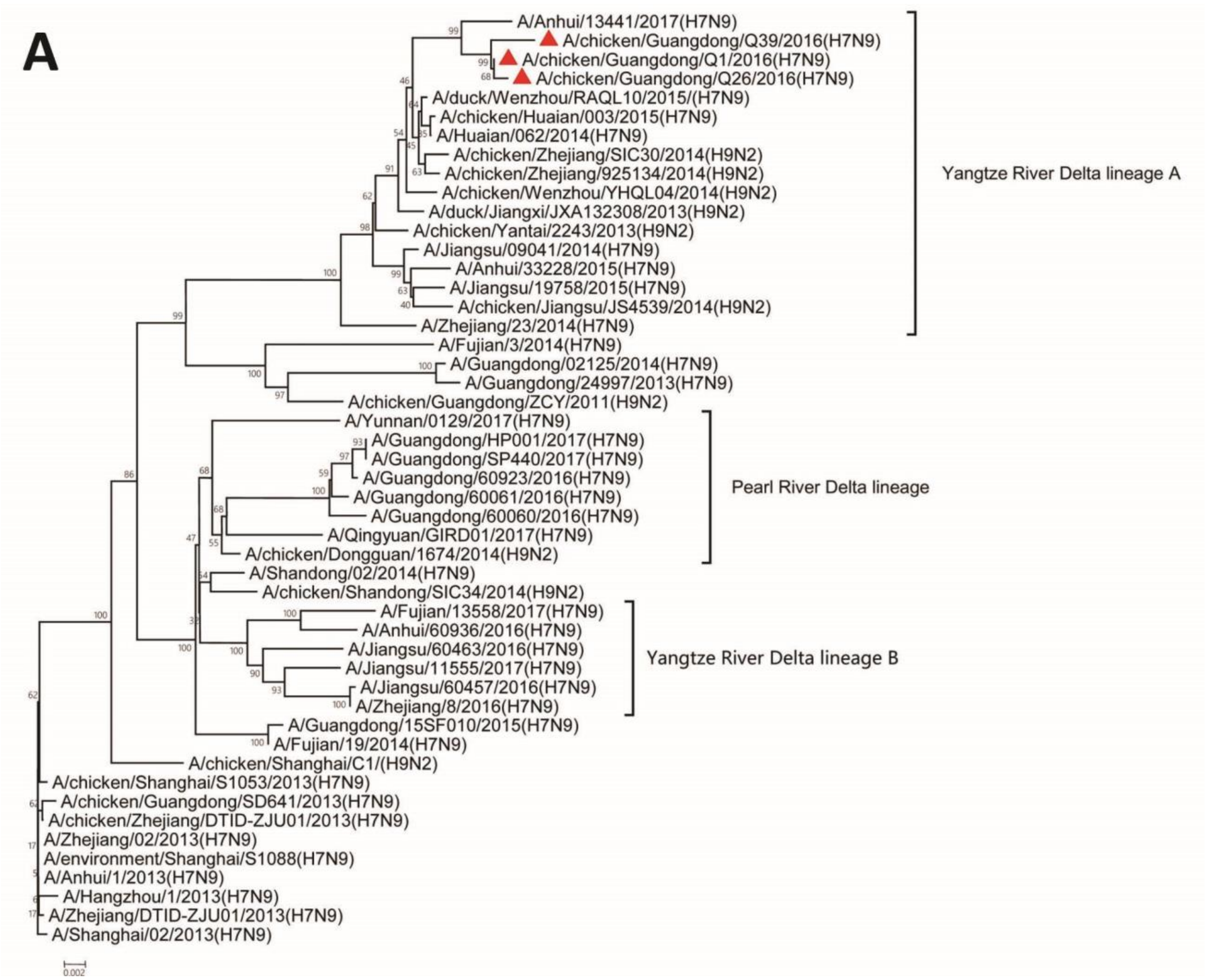
A
A/Anhui/13441/2017(H7N9)
A/chicken/Guangdong/Q39/2016(H7N9)
A/chicken/Guangdong/Q1/2016(H7N9)
A/chicken/Guangdong/Q26/2016(H7N9)
A/duck/Wenzhou/RAQL10/2015/(H7N9)
A/chicken/Huaian/003/2015(H7N9)
A/Huaian/062/2014(H7N9)
A/chicken/Zhejiang/SIC30/2014(H9N2)
A/chicken/Zhejiang/925134/2014(H9N2)
A/chicken/Wenzhou/YHQL04/2014(H9N2)
A/duck/Jiangxi/JXA132308/2013(H9N2)
A/chicken/Yantai/2243/2013(H9N2)
A/Jiangsu/09041/2014(H7N9)
A/Anhui/33228/2015(H7N9)
A/Jiangsu/19758/2015(H7N9)
A/chicken/Jiangsu/JS4539/2014(H9N2)
A/Zhejiang/23/2014(H7N9)
Yangtze River Delta lineage A
A/Fujian/3/2014(H7N9)
A/Guangdong/02125/2014(H7N9)
A/Guangdong/24997/2013(H7N9)
A/chicken/Guangdong/ZCY/2011(H9N2)
A/Yunnan/0129/2017(H7N9)
A/Guangdong/HP001/2017(H7N9)
A/Guangdong/SP440/2017(H7N9)
A/Guangdong/60923/2016(H7N9)
A/Guangdong/60061/2016(H7N9)
A/Guangdong/60060/2016(H7N9)
A/Qingyuan/GIRD01/2017(H7N9)
A/chicken/Dongguan/1674/2014(H9N2)
Pearl River Delta lineage
A/Shandong/02/2014(H7N9)
A/chicken/Shandong/SIC34/2014(H9N2)
A/Fujian/13558/2017(H7N9)
A/Anhui/60936/2016(H7N9)
A/Jiangsu/60463/2016(H7N9)
A/Jiangsu/11555/2017(H7N9)
A/Jiangsu/60457/2016(H7N9)
A/Zhejiang/8/2016(H7N9)
Yangtze River Delta lineage B
A/Guangdong/15SF010/2015(H7N9)
A/Fujian/19/2014(H7N9)
A/chicken/Shanghai/C1/(H9N2)
A/chicken/Shanghai/S1053/2013(H7N9)
A/chicken/Guangdong/SD641/2013(H7N9)
A/chicken/Zhejiang/DTID-ZJU01/2013(H7N9)
A/Zhejiang/02/2013(H7N9)
A/environment/Shanghai/S1088(H7N9)
A/Anhui/1/2013(H7N9)
A/Hangzhou/1/2013(H7N9)
A/Zhejiang/DTID-ZJU01/2013(H7N9)
A/Shanghai/02/2013(H7N9)
0.002

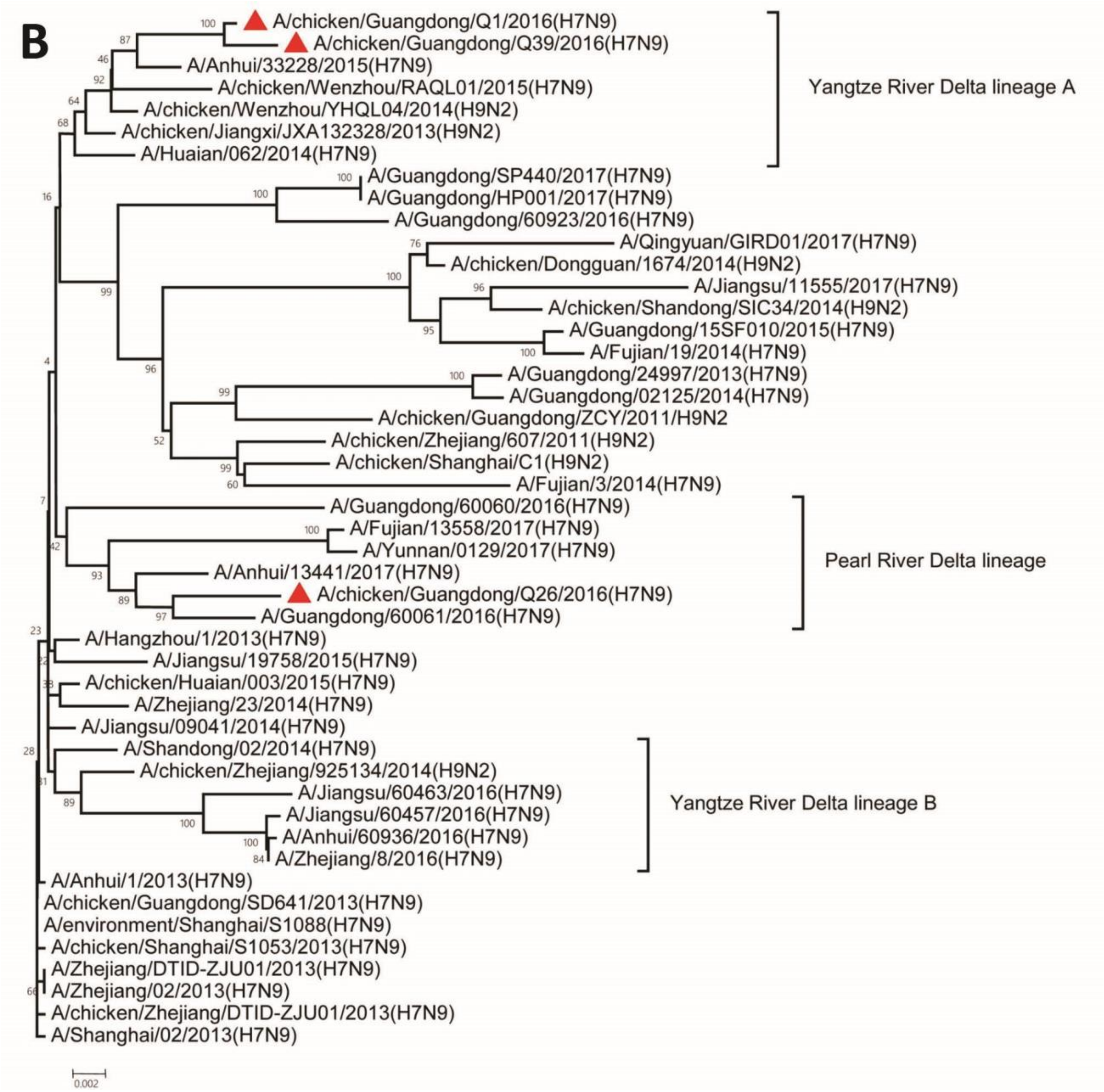
B
A/chicken/Guangdong/Q1/2016(H7N9)
A/chicken/Guangdong/Q39/2016(H7N9)
A/Anhui/33228/2015(H7N9)
A/chicken/Wenzhou/RAQL01/2015(H7N9)
A/chicken/Wenzhou/YHQL04/2014(H9N2)
A/chicken/Jiangxi/JXA132328/2013(H9N2)
A/Huaian/062/2014(H7N9)
Yangtze River Delta lineage A
A/Guangdong/SP440/2017(H7N9)
A/Guangdong/HP001/2017(H7N9)
A/Guangdong/60923/2016(H7N9)
A/Qingyuan/GIRD01/2017(H7N9)
A/chicken/Dongguan/1674/2014(H9N2)
A/Jiangsu/11555/2017(H7N9)
A/chicken/Shandong/SIC34/2014(H9N2)
A/Guangdong/15SF010/2015(H7N9)
A/Fujian/19/2014(H7N9)
A/Guangdong/24997/2013(H7N9)
A/Guangdong/02125/2014(H7N9)
A/chicken/Guangdong/ZCY/2011/H9N2
A/chicken/Zhejiang/607/2011(H9N2)
A/chicken/Shanghai/C1(H9N2)
A/Fujian/3/2014(H7N9)
A/Guangdong/60060/2016(H7N9)
A/Fujian/13558/2017(H7N9)
A/Yunnan/0129/2017(H7N9)
A/Anhui/13441/2017(H7N9)
A/chicken/Guangdong/Q26/2016(H7N9)
A/Guangdong/60061/2016(H7N9)
Pearl River Delta lineage
A/Hangzhou/1/2013(H7N9)
A/Jiangsu/19758/2015(H7N9)
A/chicken/Huaian/003/2015(H7N9)
A/Zhejiang/23/2014(H7N9)
A/Jiangsu/09041/2014(H7N9)
A/Shandong/02/2014(H7N9)
A/chicken/Zhejiang/925134/2014(H9N2)
A/Jiangsu/60463/2016(H7N9)
A/Jiangsu/60457/2016(H7N9)
A/Anhui/60936/2016(H7N9)
A/Zhejiang/8/2016(H7N9)
Yangtze River Delta lineage B
A/Anhui/1/2013(H7N9)
A/chicken/Guangdong/SD641/2013(H7N9)
A/environment/Shanghai/S1088(H7N9)
A/chicken/Shanghai/S1053/2013(H7N9)
A/Zhejiang/DTID-ZJU01/2013(H7N9)
A/Zhejiang/02/2013(H7N9)
A/chicken/Zhejiang/DTID-ZJU01/2013(H7N9)
A/Shanghai/02/2013(H7N9)
0.002

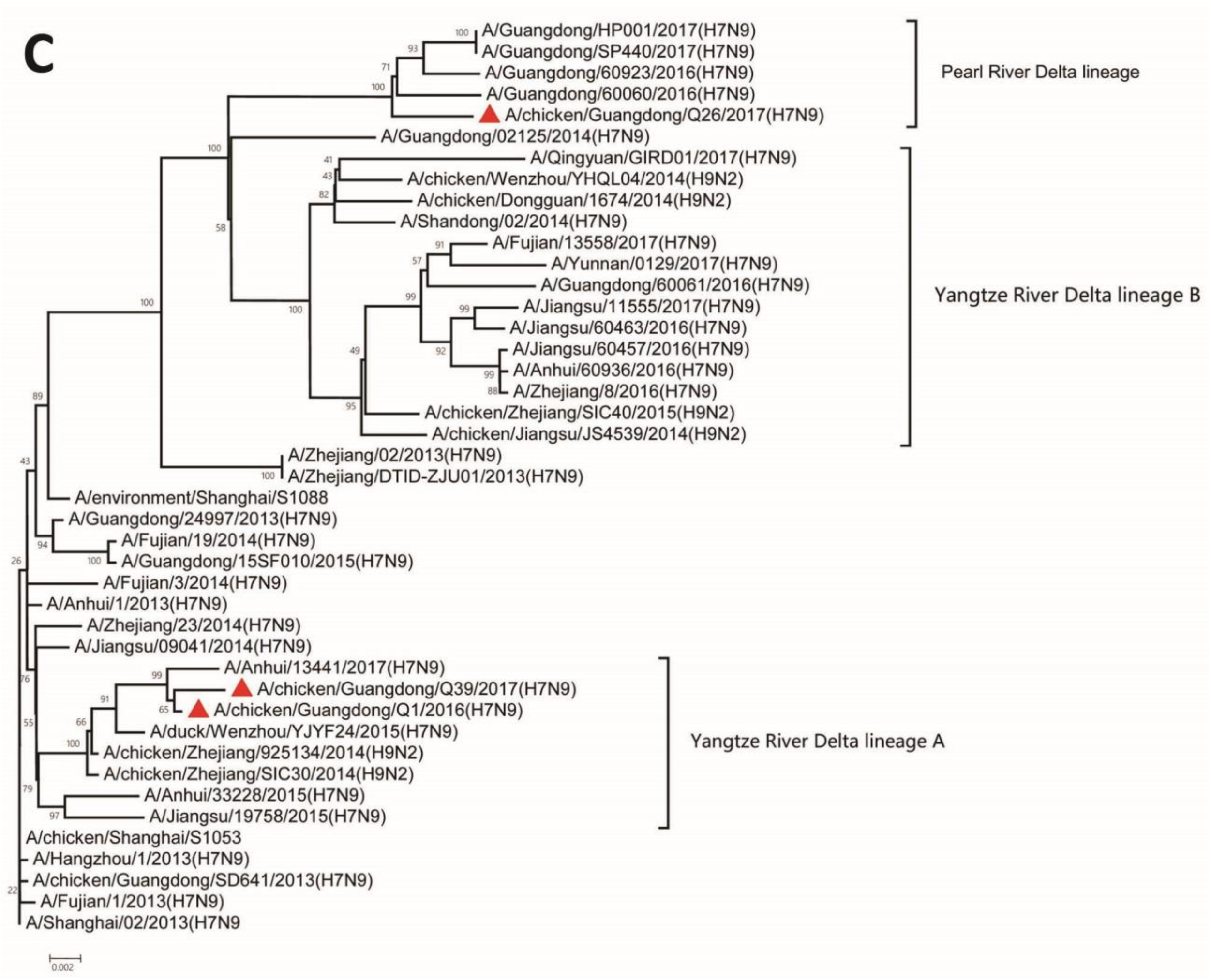
C
A/Guangdong/HP001/2017(H7N9)
A/Guangdong/SP440/2017(H7N9)
A/Guangdong/60923/2016(H7N9)
A/Guangdong/60060/2016(H7N9)
A/chicken/Guangdong/Q26/2017(H7N9)
Pearl River Delta lineage
A/Guangdong/02125/2014(H7N9)
A/Qingyuan/GIRD01/2017(H7N9)
A/chicken/Wenzhou/YHQL04/2014(H9N2)
A/chicken/Dongguan/1674/2014(H9N2)
A/Shandong/02/2014(H7N9)
A/Fujian/13558/2017(H7N9)
A/Yunnan/0129/2017(H7N9)
A/Guangdong/60061/2016(H7N9)
A/Jiangsu/11555/2017(H7N9)
A/Jiangsu/60463/2016(H7N9)
A/Jiangsu/60457/2016(H7N9)
A/Anhui/60936/2016(H7N9)
A/Zhejiang/8/2016(H7N9)
A/chicken/Zhejiang/SIC40/2015(H9N2)
A/chicken/Jiangsu/JS4539/2014(H9N2)
Yangtze River Delta lineage B
A/Zhejiang/02/2013(H7N9)
A/Zhejiang/DTID-ZJU01/2013(H7N9)
A/environment/Shanghai/S1088
A/Guangdong/24997/2013(H7N9)
A/Fujian/19/2014(H7N9)
A/Guangdong/15SF010/2015(H7N9)
A/Fujian/3/2014(H7N9)
A/Anhui/1/2013(H7N9)
A/Zhejiang/23/2014(H7N9)
A/Jiangsu/09041/2014(H7N9)
A/Anhui/13441/2017(H7N9)
A/chicken/Guangdong/Q39/2017(H7N9)
A/chicken/Guangdong/Q1/2016(H7N9)
A/duck/Wenzhou/YJYF24/2015(H7N9)
A/chicken/Zhejiang/925134/2014(H9N2)
A/chicken/Zhejiang/SIC30/2014(H9N2)
A/Anhui/33228/2015(H7N9)
A/Jiangsu/19758/2015(H7N9)
Yangtze River Delta lineage A
A/chicken/Shanghai/S1053
A/Hangzhou/1/2013(H7N9)
A/chicken/Guangdong/SD641/2013(H7N9)
A/Fujian/1/2013(H7N9)
A/Shanghai/02/2013(H7N9
0.002

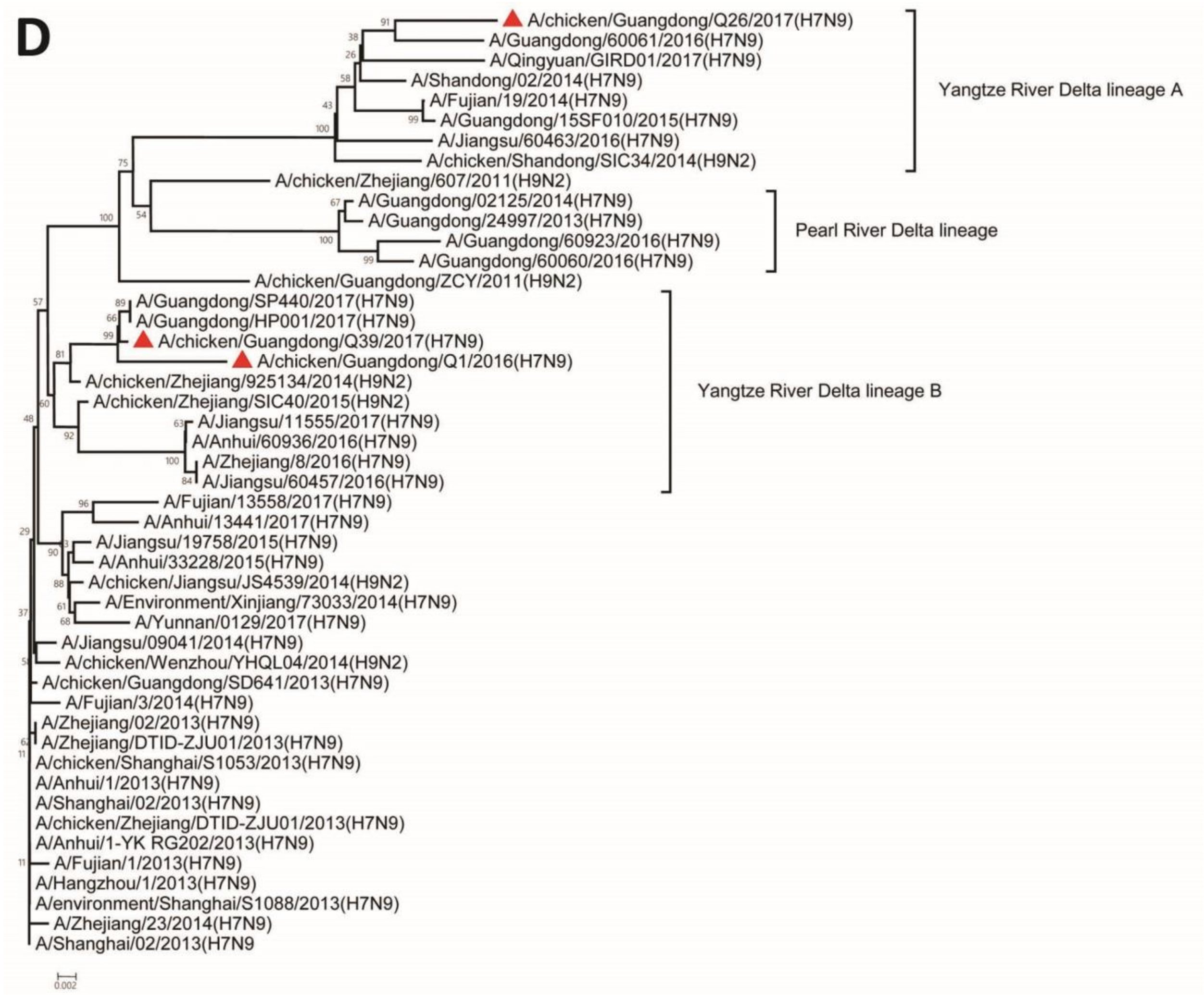
D
A/chicken/Guangdong/Q26/2017(H7N9)
A/Guangdong/60061/2016(H7N9)
A/Qingyuan/GIRD01/2017(H7N9)
A/Shandong/02/2014(H7N9)
A/Fujian/19/2014(H7N9)
A/Guangdong/15SF010/2015(H7N9)
A/Jiangsu/60463/2016(H7N9)
A/chicken/Shandong/SIC34/2014(H9N2)
Yangtze River Delta lineage A
A/chicken/Zhejiang/607/2011(H9N2)
A/Guangdong/02125/2014(H7N9)
A/Guangdong/24997/2013(H7N9)
A/Guangdong/60923/2016(H7N9)
A/Guangdong/60060/2016(H7N9)
Pearl River Delta lineage
A/chicken/Guangdong/ZCY/2011(H9N2)
A/Guangdong/SP440/2017(H7N9)
A/Guangdong/HP001/2017(H7N9)
A/chicken/Guangdong/Q39/2017(H7N9)
A/chicken/Guangdong/Q1/2016(H7N9)
A/chicken/Zhejiang/925134/2014(H9N2)
A/chicken/Zhejiang/SIC40/2015(H9N2)
A/Jiangsu/11555/2017(H7N9)
A/Anhui/60936/2016(H7N9)
A/Zhejiang/8/2016(H7N9)
A/Jiangsu/60457/2016(H7N9)
Yangtze River Delta lineage B
A/Fujian/13558/2017(H7N9)
A/Anhui/13441/2017(H7N9)
A/Jiangsu/19758/2015(H7N9)
A/Anhui/33228/2015(H7N9)
A/chicken/Jiangsu/JS4539/2014(H9N2)
A/Environment/Xinjiang/73033/2014(H7N9)
A/Yunnan/0129/2017(H7N9)
A/Jiangsu/09041/2014(H7N9)
A/chicken/Wenzhou/YHQL04/2014(H9N2)
A/chicken/Guangdong/SD641/2013(H7N9)
A/Fujian/3/2014(H7N9)
A/Zhejiang/02/2013(H7N9)
A/Zhejiang/DTID-ZJU01/2013(H7N9)
A/chicken/Shanghai/S1053/2013(H7N9)
A/Anhui/1/2013(H7N9)
A/Shanghai/02/2013(H7N9)
A/chicken/Zhejiang/DTID-ZJU01/2013(H7N9)
A/Anhui/1-YK RG202/2013(H7N9)
A/Fujian/1/2013(H7N9)
A/Hangzhou/1/2013(H7N9)
A/environment/Shanghai/S1088/2013(H7N9)
A/Zhejiang/23/2014(H7N9)
A/Shanghai/02/2013(H7N9
0.002

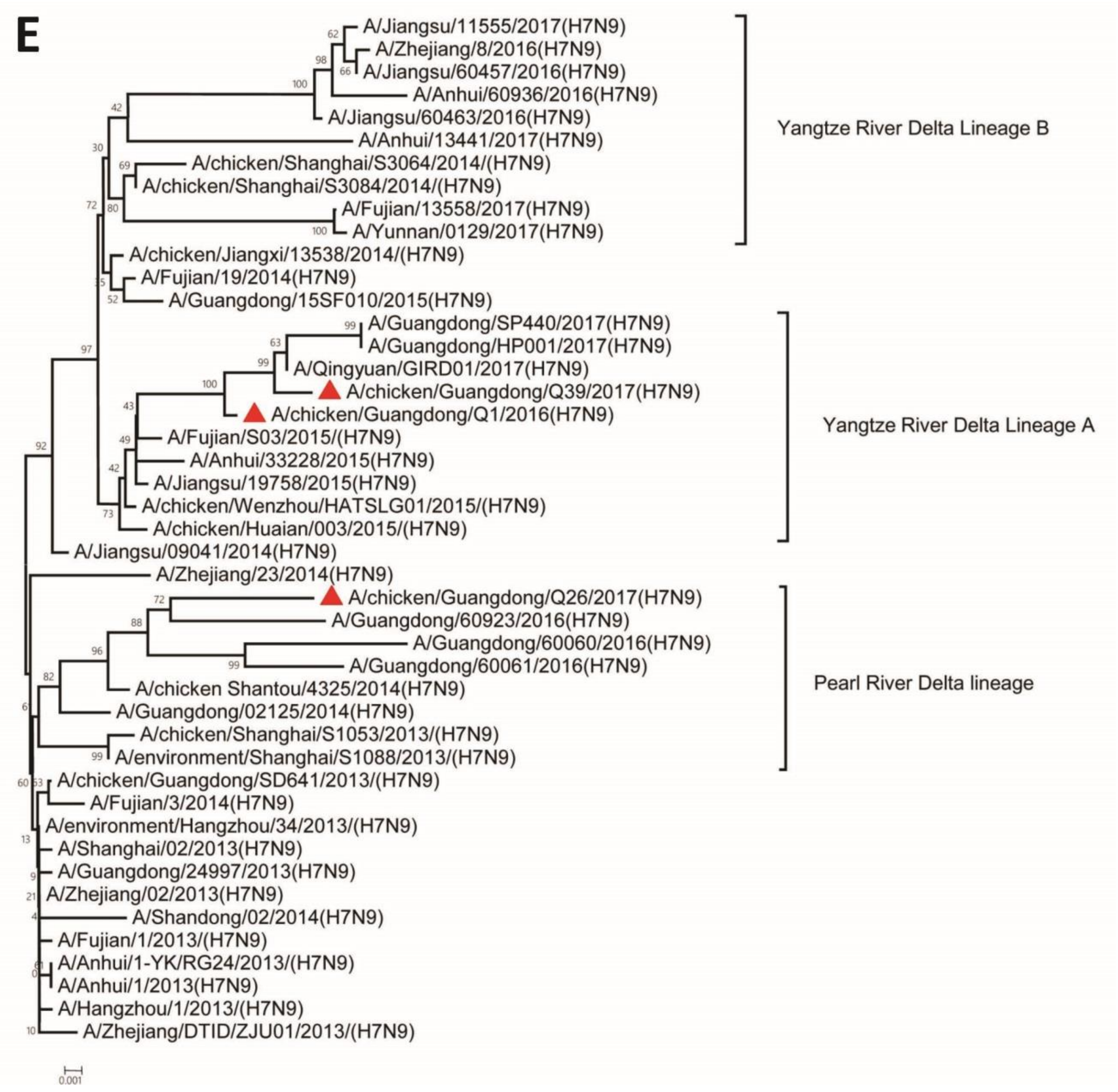
E
A/Jiangsu/11555/2017(H7N9)
A/Zhejiang/8/2016(H7N9)
A/Jiangsu/60457/2016(H7N9)
A/Anhui/60936/2016(H7N9)
A/Jiangsu/60463/2016(H7N9)
A/Anhui/13441/2017(H7N9)
A/chicken/Shanghai/S3064/2014/(H7N9)
A/chicken/Shanghai/S3084/2014/(H7N9)
A/Fujian/13558/2017(H7N9)
A/Yunnan/0129/2017(H7N9)
Yangtze River Delta Lineage B
A/chicken/Jiangxi/13538/2014/(H7N9)
A/Fujian/19/2014(H7N9)
A/Guangdong/15SF010/2015(H7N9)
A/Guangdong/SP440/2017(H7N9)
A/Guangdong/HP001/2017(H7N9)
A/Qingyuan/GIRD01/2017(H7N9)
A/chicken/Guangdong/Q39/2017(H7N9)
A/chicken/Guangdong/Q1/2016(H7N9)
A/Fujian/S03/2015/(H7N9)
A/Anhui/33228/2015(H7N9)
A/Jiangsu/19758/2015(H7N9)
A/chicken/Wenzhou/HATSLG01/2015/(H7N9)
A/chicken/Huaian/003/2015/(H7N9)
Yangtze River Delta Lineage A
A/Jiangsu/09041/2014(H7N9)
A/Zhejiang/23/2014(H7N9)
A/chicken/Guangdong/Q26/2017(H7N9)
A/Guangdong/60923/2016(H7N9)
A/Guangdong/60060/2016(H7N9)
A/Guangdong/60061/2016(H7N9)
A/chicken Shantou/4325/2014(H7N9)
A/Guangdong/02125/2014(H7N9)
A/chicken/Shanghai/S1053/2013/(H7N9)
A/environment/Shanghai/S1088/2013/(H7N9)
Pearl River Delta lineage
A/chicken/Guangdong/SD641/2013/(H7N9)
A/Fujian/3/2014(H7N9)
A/environment/Hangzhou/34/2013/(H7N9)
A/Shanghai/02/2013(H7N9)
A/Guangdong/24997/2013(H7N9)
A/Zhejiang/02/2013(H7N9)
A/Shandong/02/2014(H7N9)
A/Fujian/1/2013/(H7N9)
A/Anhui/1-YK/RG24/2013/(H7N9)
A/Anhui/1/2013(H7N9)
A/Hangzhou/1/2013/(H7N9)
A/Zhejiang/DTID/ZJU01/2013/(H7N9)
0.001

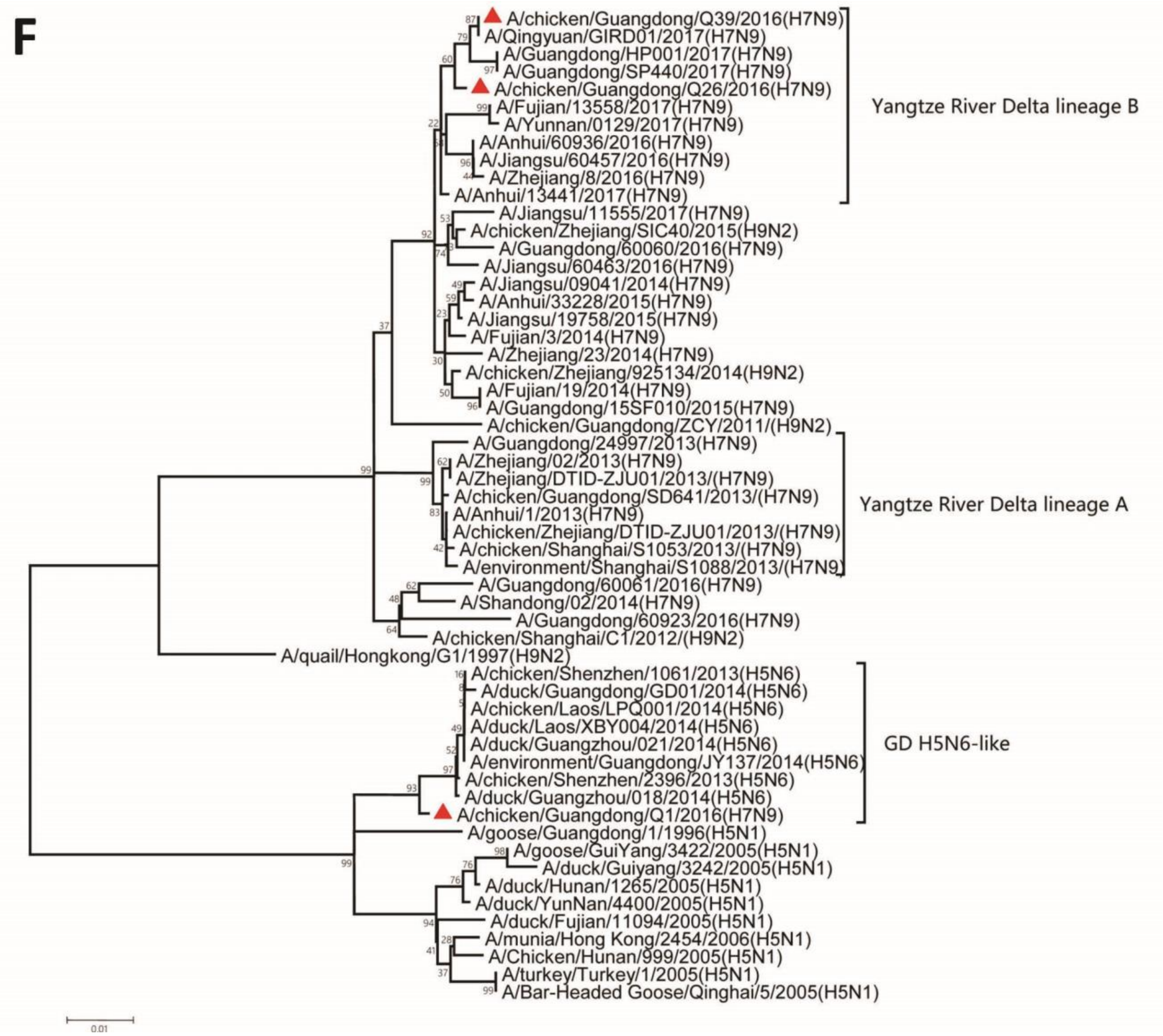
F
A/chicken/Guangdong/Q39/2016(H7N9)
A/Qingyuan/GIRD01/2017(H7N9)
A/Guangdong/HP001/2017(H7N9)
A/Guangdong/SP440/2017(H7N9)
A/chicken/Guangdong/Q26/2016(H7N9)
A/Fujian/13558/2017(H7N9)
A/Yunnan/0129/2017(H7N9)
A/Anhui/60936/2016(H7N9)
A/Jiangsu/60457/2016(H7N9)
A/Zhejiang/8/2016(H7N9)
A/Anhui/13441/2017(H7N9)
Yangtze River Delta lineage B
A/Jiangsu/11555/2017(H7N9)
A/chicken/Zhejiang/SIC40/2015(H9N2)
A/Guangdong/60060/2016(H7N9)
A/Jiangsu/60463/2016(H7N9)
A/Jiangsu/09041/2014(H7N9)
A/Anhui/33228/2015(H7N9)
A/Jiangsu/19758/2015(H7N9)
A/Fujian/3/2014(H7N9)
A/Zhejiang/23/2014(H7N9)
A/chicken/Zhejiang/925134/2014(H9N2)
A/Fujian/19/2014(H7N9)
A/Guangdong/15SF010/2015(H7N9)
A/chicken/Guangdong/ZCY/2011/(H9N2)
A/Guangdong/24997/2013(H7N9)
A/Zhejiang/02/2013(H7N9)
A/Zhejiang/DTID-ZJU01/2013/(H7N9)
A/chicken/Guangdong/SD641/2013/(H7N9)
A/Anhui/1/2013(H7N9)
A/chicken/Zhejiang/DTID-ZJU01/2013/(H7N9)
A/chicken/Shanghai/S1053/2013/(H7N9)
A/environment/Shanghai/S1088/2013/(H7N9)
Yangtze River Delta lineage A
A/Guangdong/60061/2016(H7N9)
A/Shandong/02/2014(H7N9)
A/Guangdong/60923/2016(H7N9)
A/chicken/Shanghai/C1/2012/(H9N2)
A/quail/Hongkong/G1/1997(H9N2)
A/chicken/Shenzhen/1061/2013(H5N6)
A/duck/Guangdong/GD01/2014(H5N6)
A/chicken/Laos/LPQ001/2014(H5N6)
A/duck/Laos/XBY004/2014(H5N6)
A/duck/Guangzhou/021/2014(H5N6)
A/environment/Guangdong/JY137/2014(H5N6)
A/chicken/Shenzhen/2396/2013(H5N6)
A/duck/Guangzhou/018/2014(H5N6)
A/chicken/Guangdong/Q1/2016(H7N9)
GD H5N6-like
A/goose/Guangdong/1/1996(H5N1)
A/goose/GuiYang/3422/2005(H5N1)
A/duck/Guiyang/3242/2005(H5N1)
A/duck/Hunan/1265/2005(H5N1)
A/duck/YunNan/4400/2005(H5N1)
A/duck/Fujian/11094/2005(H5N1)
A/munia/Hong Kong/2454/2006(H5N1)
A/Chicken/Hunan/999/2005(H5N1)
A/turkey/Turkey/1/2005(H5N1)
A/Bar-Headed Goose/Qinghai/5/2005(H5N1)
0.01

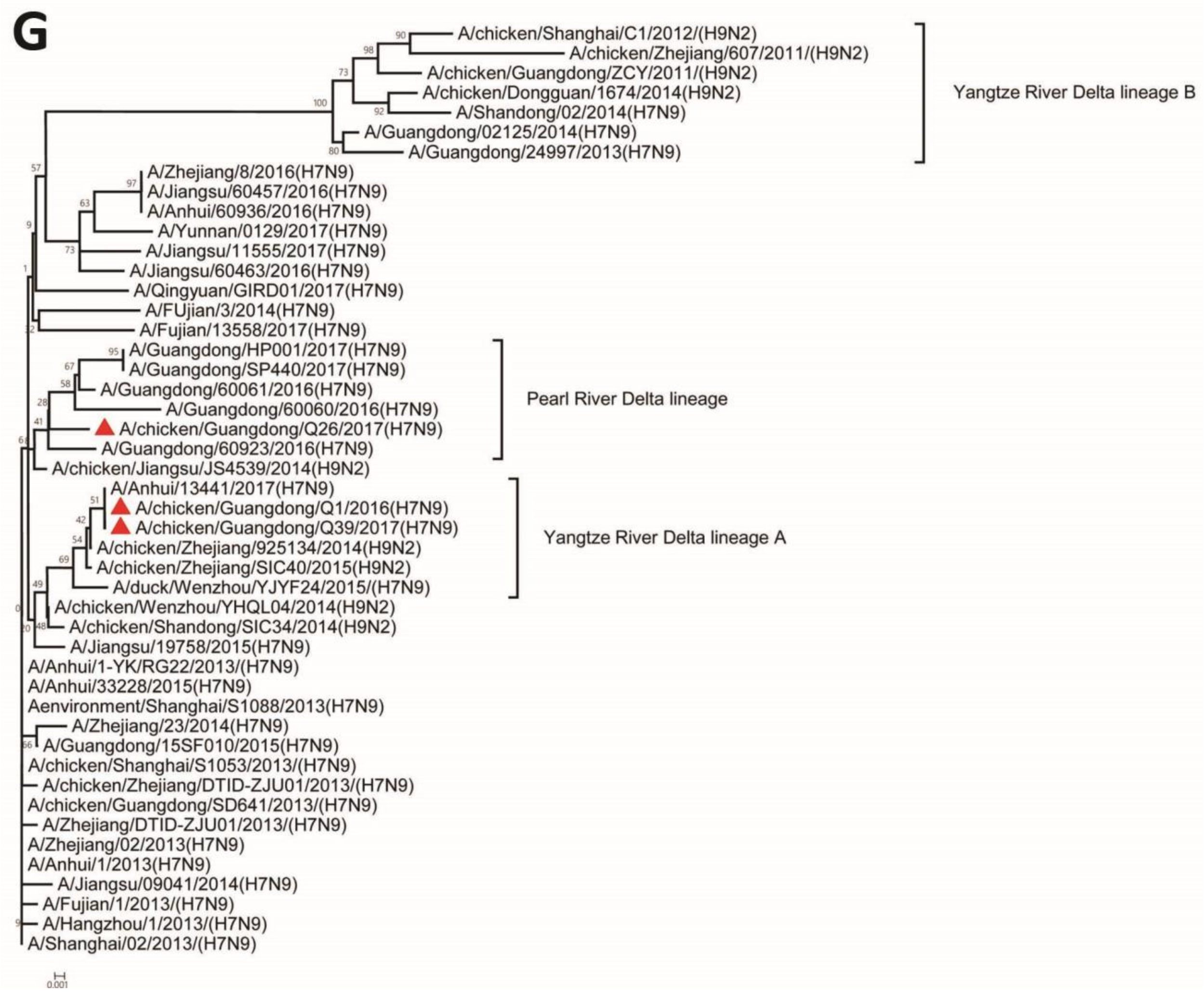

Technical Appendix Figure. Phylogenetic analyses of 3 isolates (triangles) of avian influenza (H7N9) virus obtained from chickens in southern China, 2016–2017, compared with reference isolates. The tree was constructed by using the neighbor-joining method with the maximum composite likelihood model in MEGA version 5.2 (http://www.megasoftware.net) with 1,000 bootstrap replicates, on the basis of the following sequences: PB2 (A): nt 28–2307; PB1 (B): nt 25–2298; PA (C): nt 25–2175; NP (D): nt46–1542; NA (E): nt 19–1416; M(F): nt26–784; NS1(G): nt 27–680. Virus lineages are shown at right. Scale bars indicates nucleotide substitutions per site.
